# Supplementary material for: Throwing and manipulating and cheating with a DNA nano-dice
Source: Nat Commun. 2023 Apr 28;14:2440. doi: 10.1038/s41467-023-38164-7 (PMC10147716; doi:10.1038/s41467-023-38164-7)
Supplement: Supplementary file 1 — Supplementary Information [file 41467_2023_38164_MOESM1_ESM.pdf]

Supplementary Information for

Throwing and manipulating and cheating with a DNA nano-dice

Xiaochen Tang[1,2#], Tianshu Chen[1,2#], Wenxing Li[3#], Dongsheng Mao[3], Chenbin Liu[3], Qi Wu[3], Nan Huang[3], Song Hu[3], Fenyong Sun[3*], Qiuhui Pan[1,2*], Xiaoli Zhu[3*]

[1] Department of Clinical Laboratory Medicine, Shanghai Children's Medical Center, School of Medicine, Shanghai Jiao Tong University, Shanghai 200127, P. R. China.

[2] Shanghai Key Laboratory of Clinical Molecular Diagnostics for Pediatrics, Shanghai 200127, P. R. China.

[3] Department of Clinical Laboratory Medicine, Shanghai Tenth People's Hospital of Tongji University, Shanghai 200072, P. R. China.

[#] These authors contributed equally to this work.

*Corresponding authors:

Fenyong Sun, Email: sunfenyong@263.net

Qiuhui Pan, Email: panqiuhui_med@163.com

Xiaoli Zhu, Email: xiaolizhu@shu.edu.cn (X.Z.)

The PDF file includes:

Supplementary Fig. 1 to 16

Supplementary Tables 1 to 6

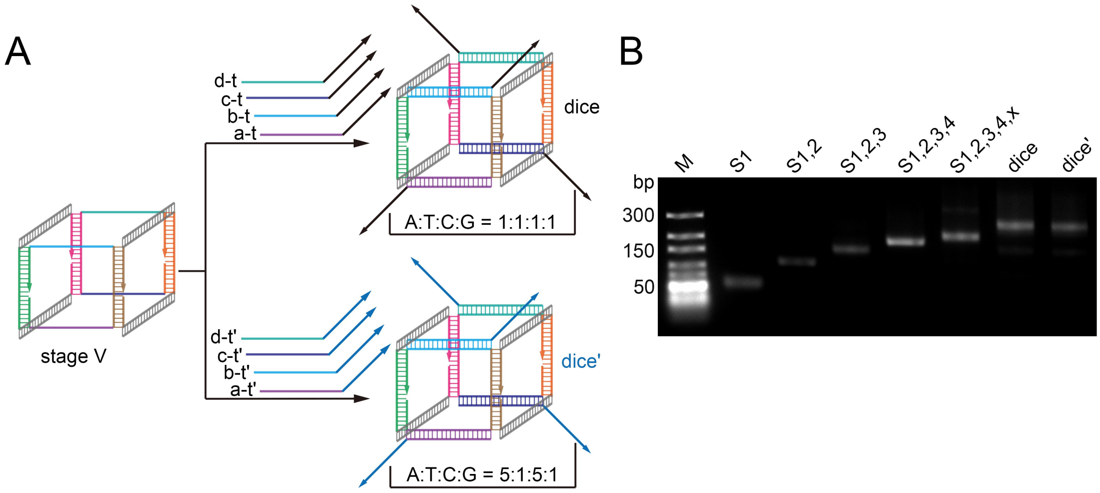

Supplementary Fig. 1. Construction and AGE analysis of dice and dice'. (A) Schematic illustration of dice assembly. (B) 2.5% AGE analysis of the stepwise assembly of dice and dice' from 3 independent experiments. The concentration of each strand is 500 nM. Source data are provided as a Source Data file.

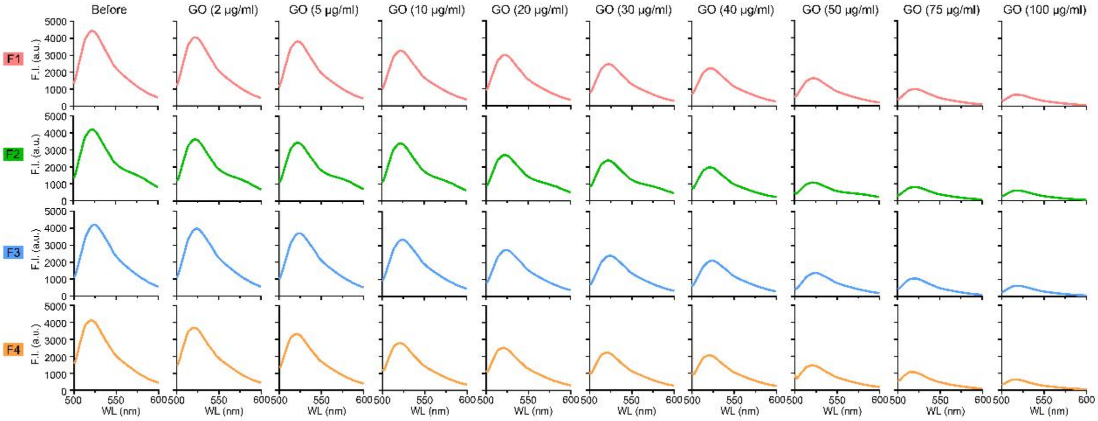

Supplementary Fig. 2. Fluorescence spectra of four signals of the nano-dice with different concentrations of GO.

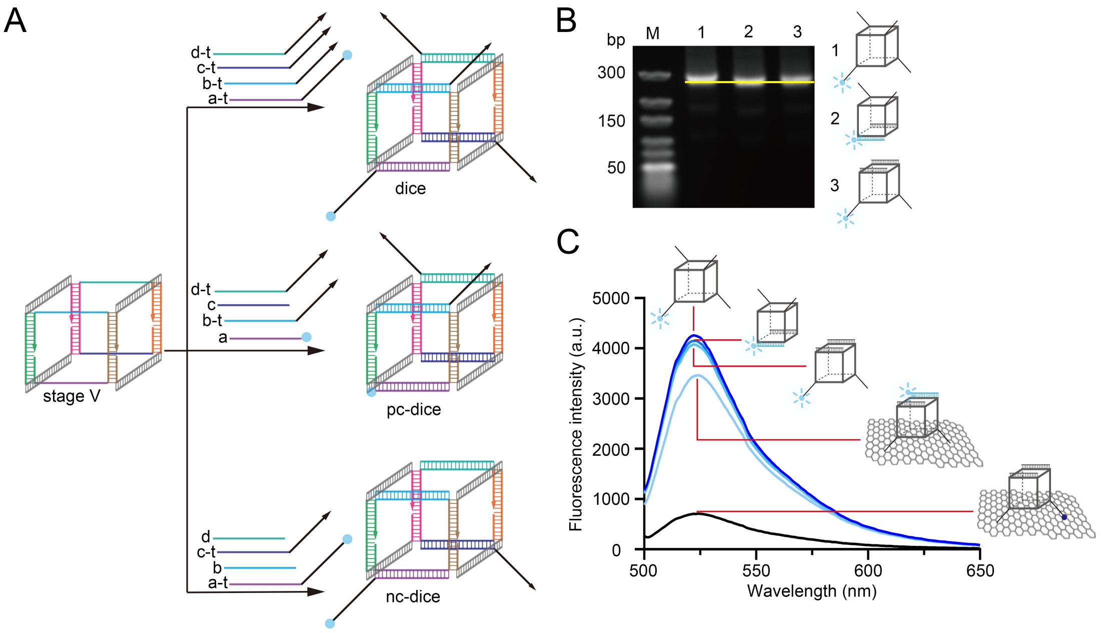

Supplementary Fig. 3. Construction and analysis of control dice. (A) Schematic illustration of control dice assembly. pc-dice is used for the correction to 1 and nc-dice is used for the correction to 0. (B) 2.5% AGE analysis of the assembly of normal and control dice from 3 independent experiments. The concentration of each strand is 500 nM. (C) Fluorescence spectral of three dice without GO and two control dice with GO. Source data are provided as a Source Data file.

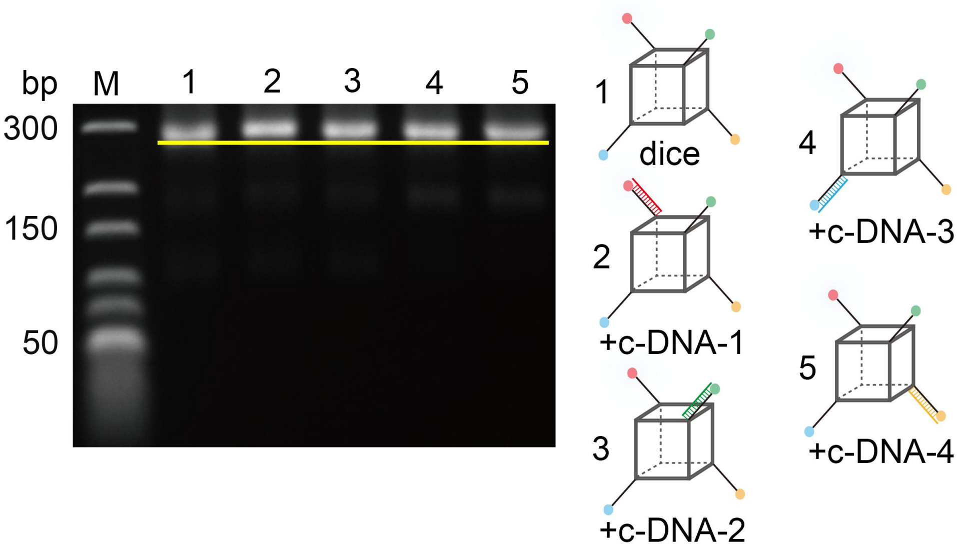

Supplementary Fig. 4. Response analysis of c-DNA to dice by 2.5% AGE from 3 independent experiments. Line 1, normal dice (500 nM); Line 2 to 5, normal dice (500 nM) mixed with c-DNA (1.5 μM) for 90 min. Source data are provided as a Source Data file.

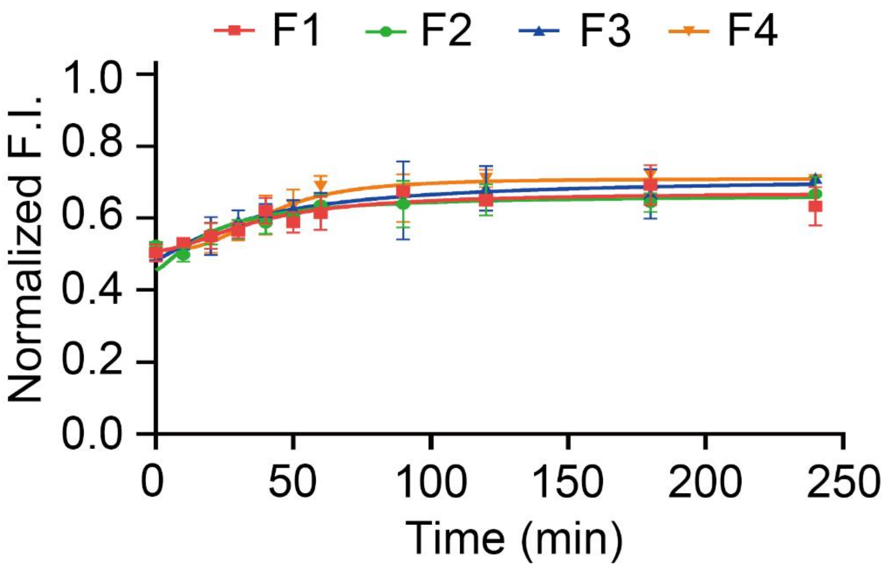

Supplementary Fig. 5. Kinetics of four signals change induced by adding the non-c-DNA. Four fluorescence signals were all increased regardless of its complementarity, n=3 independent experiments. Data are presented as mean values ± s.d. Source data are provided as a Source Data file.

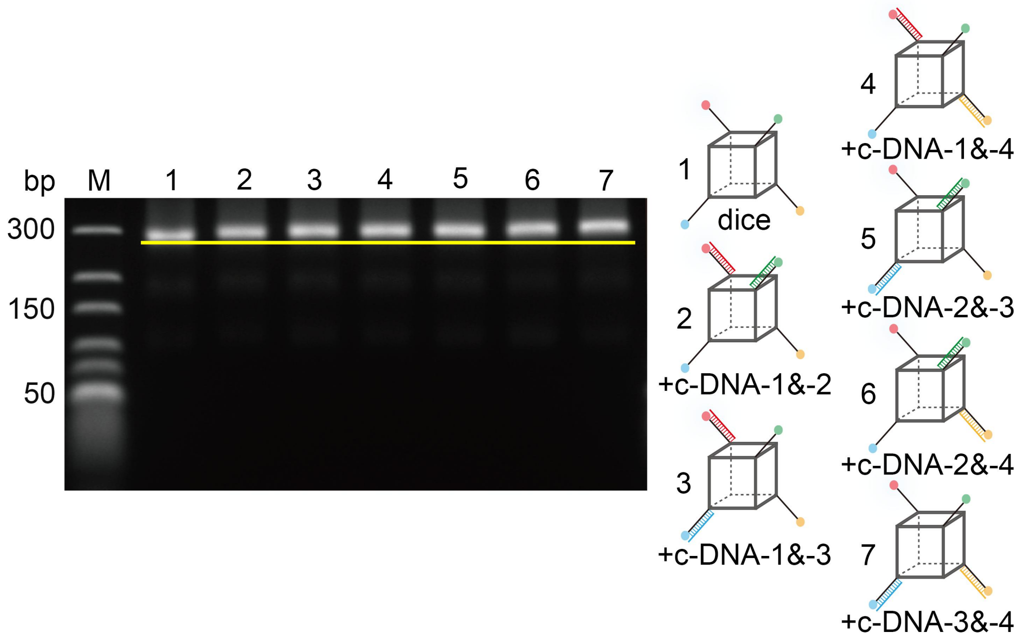

Supplementary Fig. 6. Response analysis of two c-DNAs to dice by 2.5% AGE from 3 independent experiments. Line 1, normal dice (500 nM); Line 2 to 7, normal dice (500 nM) mixed with two c-DNAs (1.5 μM) for 90 min. Source data are provided as a Source Data file.

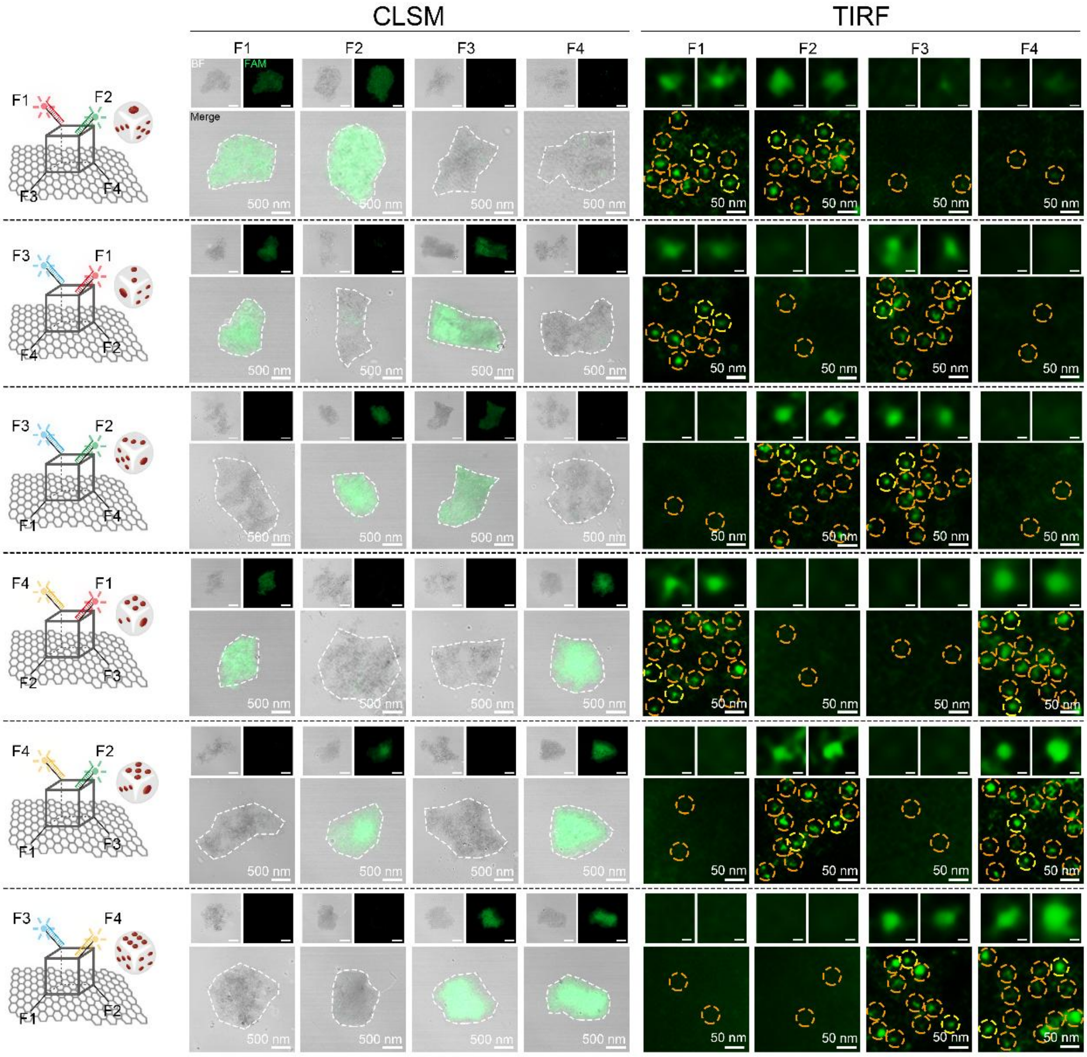

Supplementary Fig. 7. Imaging characterization of four signals in the artificial manipulation with two c-DNAs to the nano-dice from 3 independent experiments. Left, confocal images of four signals in the dice with two c-DNAs. The scale bars are 500 nm in split and merge images. Right, single-molecule images of four signals in dice with two c-DNAs. The scale bars are 50 nm in original images and 10 nm in enlarged images, respectively. The bright spots in yellow circle were enlarged to show.

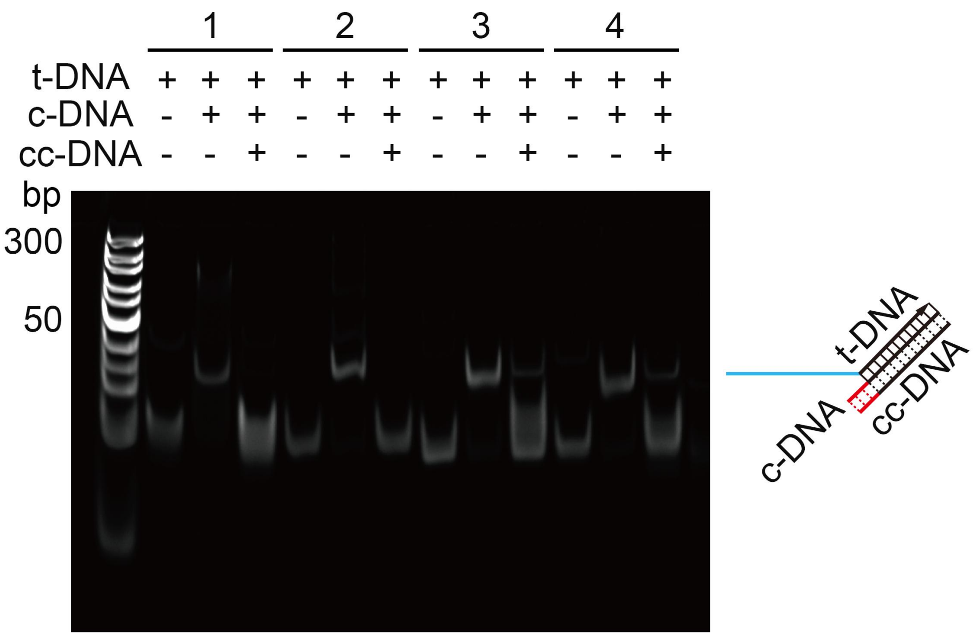

Supplementary Fig. 8. Native-PAGE analysis of SDRs from 3 independent experiments. 20% native-PAGE analysis of SDRs among c-DNA, t-DNA and cc-DNA. The concentration of each strand is 1 μM. Source data are provided as a Source Data file.

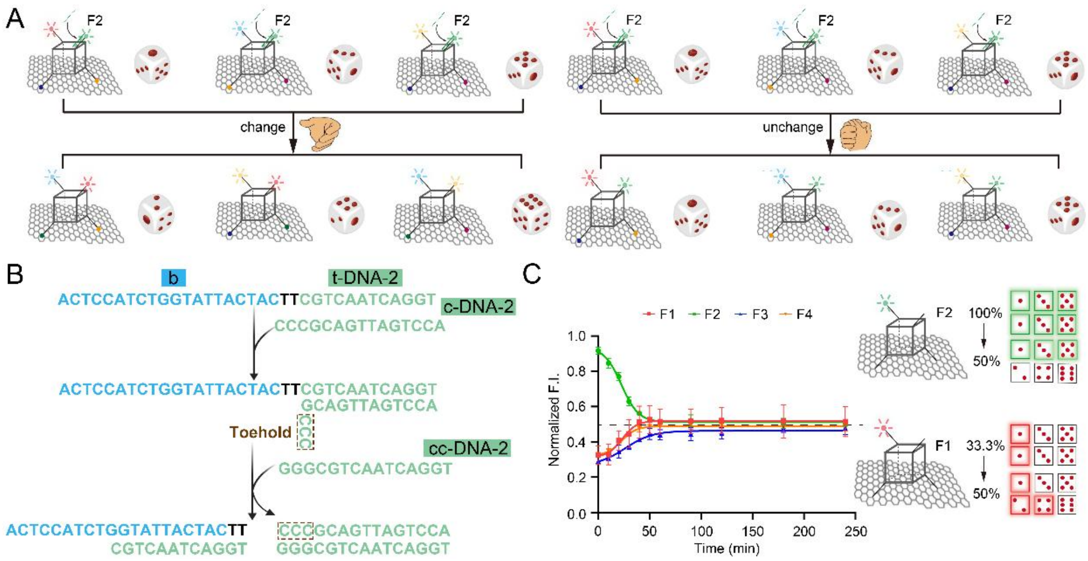

Supplementary Fig. 9. Artificial manipulation of the DNA nano-dice system with cc-DNA-2. (A) Schematic illustration of cc-DNA-2 adding to DNA nano-dice system. (B) Schematic illustration of SDR among t-DNA-2, c-DNA-2, and cc-DNA-2. (C) Kinetics of four signals change induced by adding the cc-DNA-2, n=3 independent experiments. Right, schematic illustration of theoretical changes in the pips and fluorescence of dice. Data are presented as mean values ± s.d. Source data are provided as a Source Data file.

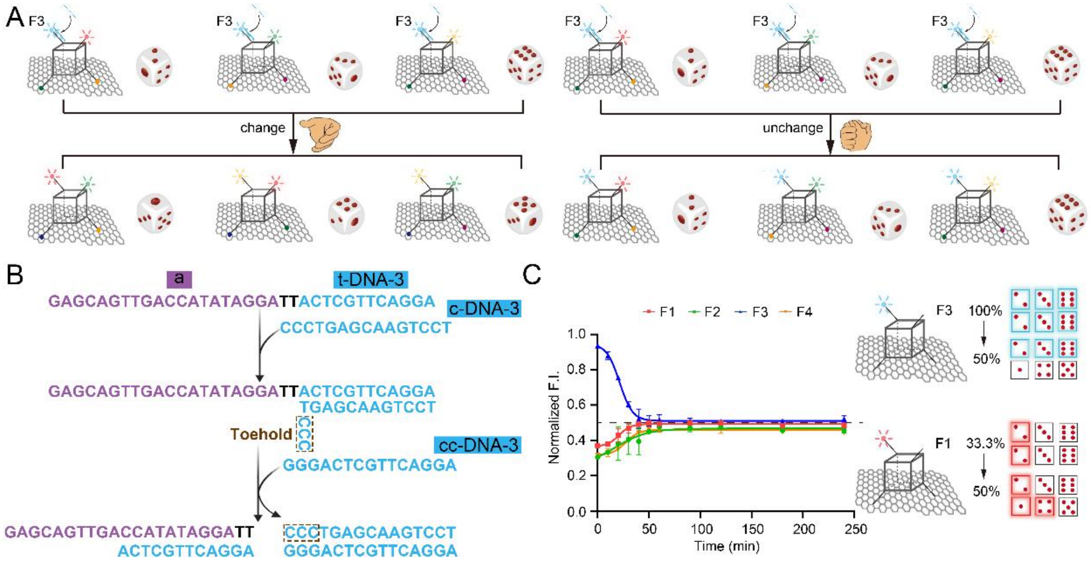

Supplementary Fig. 10. Artificial manipulation of the DNA nano-dice system with cc-DNA-3. (A) Schematic illustration of cc-DNA-3 adding to DNA nano-dice system. (B) Schematic illustration of SDR among t-DNA-3, c-DNA-3, and cc-DNA-3. (C) Kinetics of four signals change induced by adding the cc-DNA-3, n=3 independent experiments. Right, schematic illustration of theoretical changes in the pips and fluorescence of dice. Data are presented as mean values ± s.d. Source data are provided as a Source Data file.

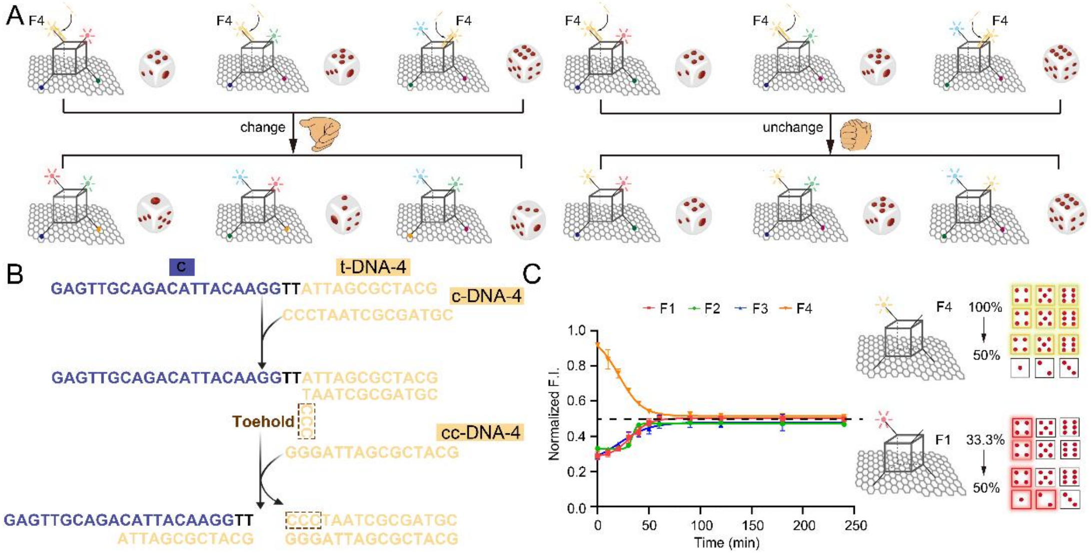

Supplementary Fig. 11. Artificial manipulation of the DNA nano-dice system with cc-DNA-4. (A) Schematic illustration of cc-DNA-4 adding to DNA nano-dice system. (B) Schematic illustration of SDR among t-DNA-4, c-DNA-4, and cc-DNA-4. (C) Kinetics of four signals change induced by adding the cc-DNA-4, n=3 independent experiments. Right, schematic illustration of theoretical changes in the pips and fluorescence of dice. Data are presented as mean values ± s.d. Source data are provided as a Source Data file.

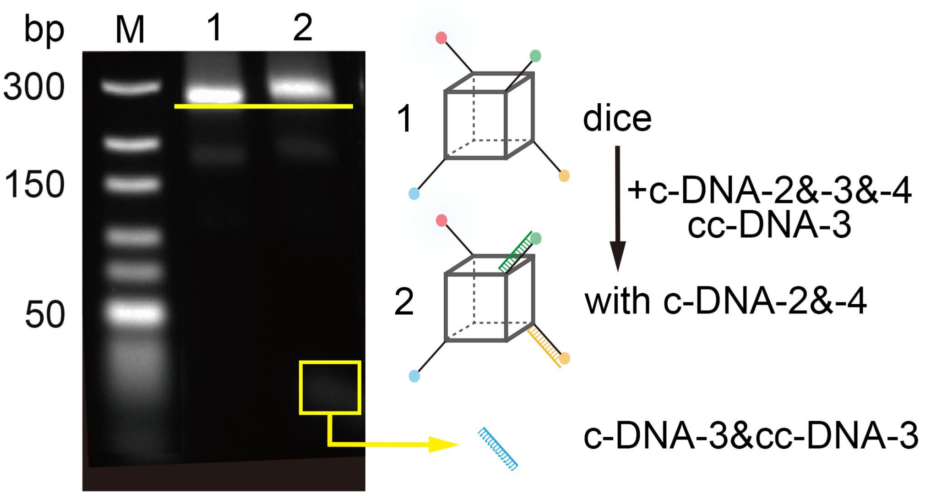

Supplementary Fig. 12. Response analysis of dice with SDRs by 2.5% AGE from 3 independent experiments. Line 2, normal dice (500 nM); Line 3, normal dice (500 nM) mixed with c-DNA-3 (1.5 μM) and c-DNA-4 (1.5 μM) for 90 min and then adding c-DNA-2 (1.5 μM) and cc-DNA-3 (1.5 μM). Source data are provided as a Source Data file.

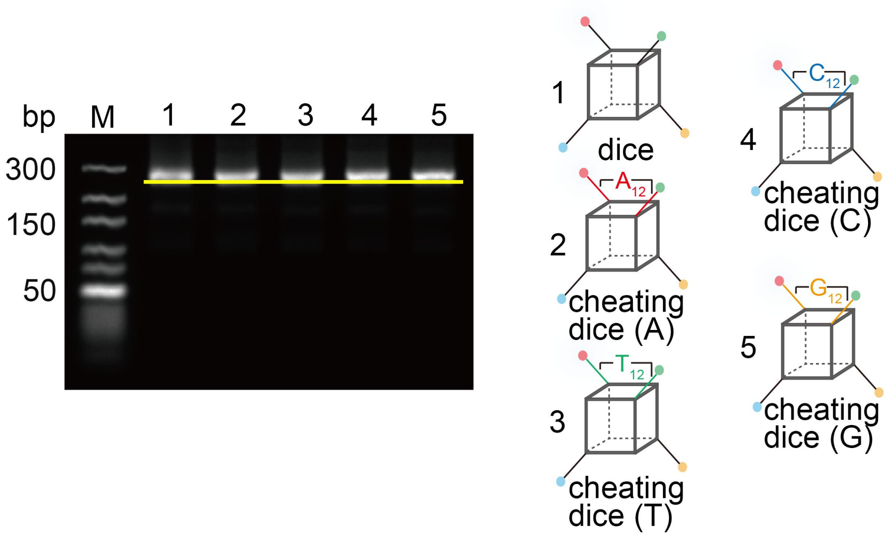

Supplementary Fig. 13. Construction and AGE analysis of cheating dice (A/T/C/G) from 3 independent experiments. 2.5% AGE analysis of the assembly of normal and cheating dice. The concentration of each strand is 500 nM. Source data are provided as a Source Data file.

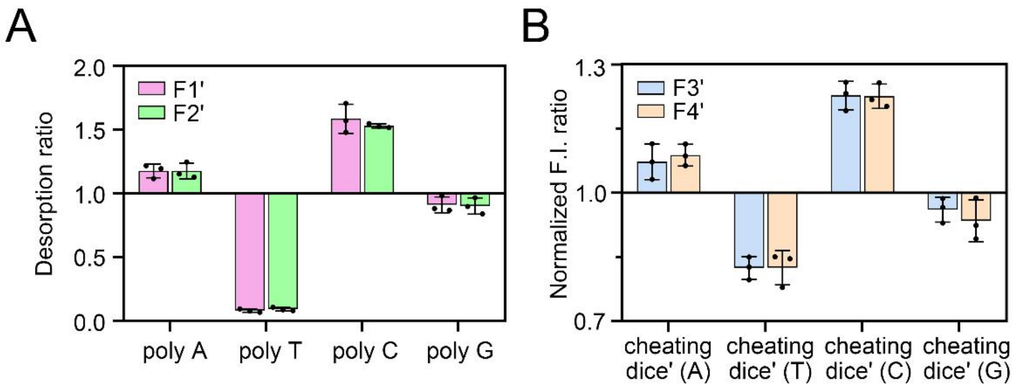

Supplementary Fig. 14. Relevant verification of DNA (group 2) and cheating dice'. (A) Desorption ratio of FAM labeled original DNA (group 2) from GO by poly $A_{12}/T_{12}/C_{12}/G_{12}$ after 60 min reaction, n=3 independent experiments. Data are presented as mean values ± s.d. (B) Normalized fluorescence intensity ratio of cheating dice' (A/T/C/G), n=3 independent experiments. Data are presented as mean values ± s.d. Source data are provided as a Source Data file.

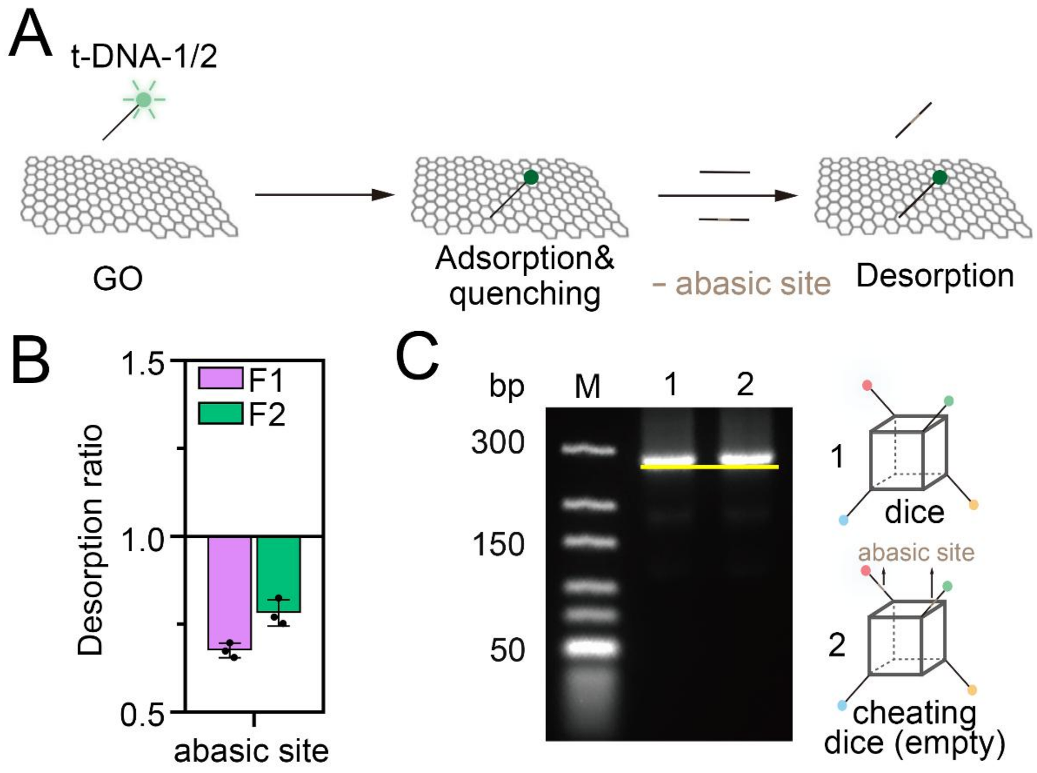

Supplementary Fig. 15. Relevant verification of modification with abasic sites. (A) Schematic illustration of adsorption and desorption of original ssDNA by adding ssDNA with abasc sites. (B) Desorption percentage of ssDNA with abasic sites, n=3 independent experiments. The percentage of DNA desorbed at 1 h after adding 1.5 μM altered DNA to FAM-labeled original DNA adsorbed on GO. Desorption ratio "1" is defined as desorption percentage of 1.5 μM original DNA to FAM-labeled original DNA adsorbed on GO. Data are presented as mean values ± s.d. (C) 2.5% AGE analysis of the assembly of normal dice and cheating dice (empty) from 3 independent experiments. The concentration of each strand is 500 nM. Source data are provided as a Source

Data file.

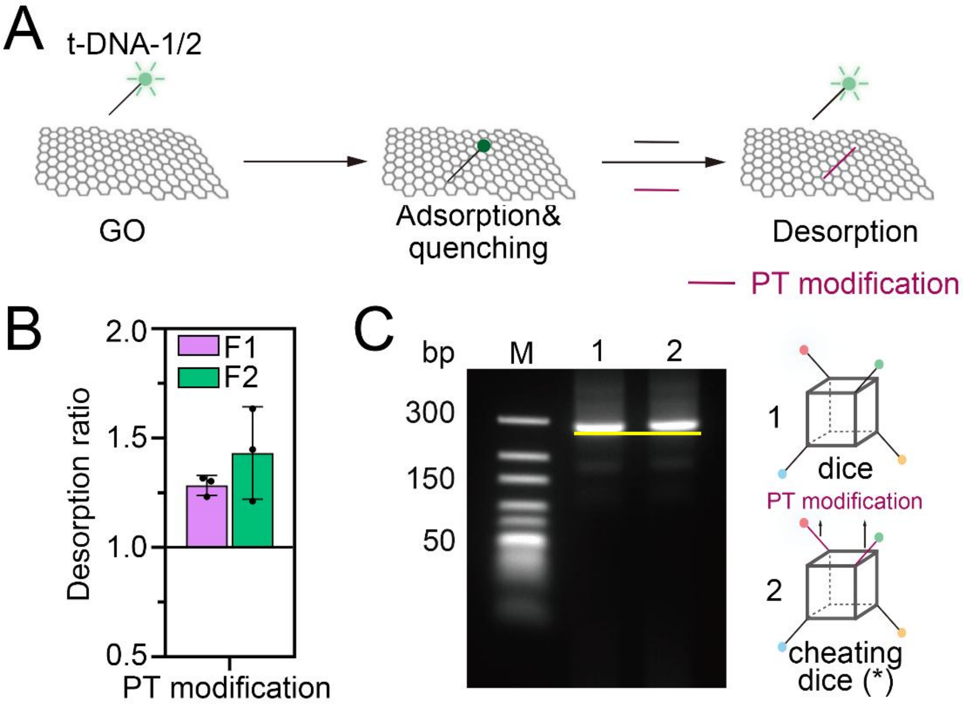

Supplementary Fig. 16. Relevant verification of phosphorothioate modification. (A) Schematic illustration of adsorption and desorption of original ssDNA by adding ssDNA with phosphorothioate modification (PT modification). (B) Desorption percentage of ssDNA with phosphorothioate modification, n=3 independent experiments. The percentage of DNA desorbed at 1 h after adding 1.5 μM altered DNA to FAM-labeled original DNA adsorbed on GO. Desorption ratio "1" is defined as desorption percentage of 1.5 μM original DNA to FAM-labeled original DNA adsorbed on GO. Data are presented as mean values ± s.d. (C) 2.5% AGE analysis of the assembly of normal dice and cheating dice (*) from 3 independent experiments. The concentration of each strand is 500 nM. Source data are provided as a Source Data file.

Supplementary Table 1. Sequences of oligonucleotides.

Strand	Sequences (5'-3')
S1	TCGCTGAGTATTTCCTATATGGTCAACTGCTCTTGCAAGTGTGGGCACGC ACACTTGTAGTAATACCAGATGGAGTTTCACAAATCTG
S2	CCACACTTGCTTCTTCCTTCCTTCCTTCCTTCTTCTGACCAGTGTCAGCA AACCTTCTTCCTTCCTTCCTTCCTTCTTGTGTGCGTGC
S3	CACTGGTCAGTTCCTTGTAATGTCTGCAACTCTTCTACCGATAGCCGCTA GTTGTTGAAATGCTGTAAGATGTACTTTGGTTTGCTGA
S4	CTATCGGTAGTTCTTCCTTCCTTCCTTCCTTCTTTACTCAGCGACAGATTT GTGTTCTTCCTTCCTTCCTTCCTTCTTCAACTAGCGG
x	GAAGGAAGGAAGGAAGGAAG
a-t	GAGCAGTTGACCATATAGGATTACTCGTTCAGGA
b-t	ACTCCATCTGGTATTACTACTTCGTCAATCAGGT
c-t	GAGTTGCAGACATTACAAGGTTATTAGCGCTACG
d-t	AGTACATCTTACAGCATTTCTTGAGTGCAATCCT
a-t'	GAGCAGTTGACCATATAGGATTGCTCAACCACAA
b-t'	ACTCCATCTGGTATTACTACTTCAGCAACCACTA
c-t'	GAGTTGCAGACATTACAAGGTTATCAGCACCACA
d-t'	AGTACATCTTACAGCATTTCTTCAGTACAACCCA
a	GAGCAGTTGACCATATAGGA
b	ACTCCATCTGGTATTACTAC
c	GAGTTGCAGACATTACAAGG
d	AGTACATCTTACAGCATTTC
a-t-FAM	GAGCAGTTGACCATATAGGATTACTCGTTCAGGA-FAM
b-t-FAM	ACTCCATCTGGTATTACTACTTCGTCAATCAGGT-FAM
c-t-FAM	GAGTTGCAGACATTACAAGGTTATTAGCGCTACG-FAM
d-t-FAM	AGTACATCTTACAGCATTTCTTGAGTGCAATCCT-FAM
a-t'-FAM	GAGCAGTTGACCATATAGGATTGCTCAACCACAA-FAM
b-t'-FAM	ACTCCATCTGGTATTACTACTTCAGCAACCACTA-FAM
c-t'-FAM	GAGTTGCAGACATTACAAGGTTATCAGCACCACA-FAM
d-t'-FAM	AGTACATCTTACAGCATTTCTTCAGTACAACCCA-FAM
a-FAM	GAGCAGTTGACCATATAGGA-FAM
t-DNA-1 (A)	AGTACATCTTACAGCATTTCTTAAAAAAAAAAAA
t-DNA-2 (A)	ACTCCATCTGGTATTACTACTTAAAAAAAAAAAA
t-DNA-1 (T)	AGTACATCTTACAGCATTTCTTTTTTTTTTTTTT
t-DNA-2 (T)	ACTCCATCTGGTATTACTACTTTTTTTTTTTTTT
t-DNA-1 (C)	AGTACATCTTACAGCATTTCTTCCCCCCCCCCCC
t-DNA-2 (C)	ACTCCATCTGGTATTACTACTTCCCCCCCCCCCC
t-DNA-1 (G)	AGTACATCTTACAGCATTTCTTGGGGGGGGGGGG
t-DNA-2 (G)	ACTCCATCTGGTATTACTACTTGGGGGGGGGGGG
t-DNA-1 (dspacer)	AGTACATCTTACAGCATTTCTTGAGTG/idSp//idSp//idSp/TCCT
t-DNA-2 (dspacer)	ACTCCATCTGGTATTACTACTTCGTCA/idSp//idSp//idSp/AGGT

t-DNA-1 (*)	AGTACATCTTACAGCATTTCTTG*A*G*T*G*C*A*A*T*C*C*T
t-DNA-2 (*)	ACTCCATCTGGTATTACTACTTC*G*T*C*A*A*T*C*A*G*G*T
c-DNA-1	AGGATTGCACTCCCC
c-DNA-2	ACCTGATTGACGCCC
c-DNA-3	TCCTGAACGAGTCCC
c-DNA-4	CGTAGCGCTAATCCC
cc-DNA-1	GGGGAGTGCAATCCT
cc-DNA-2	GGGCGTCAATCAGGT
cc-DNA-3	GGGACTCGTTCAGGA
cc-DNA-4	GGGATTAGCGCTACG

Supplementary Table 2. Description of four signals in Fig. 5C.

	c-DNA-1	c-DNA-2	c-DNA-3	c-DNA-4
F1	0.908±0.021	0.331±0.011	0.321±0.011	0.315±0.026
F2	0.299±0.038	0.888±0.025	0.303±0.006	0.318±0.04
F3	0.329±0.009	0.323±0.02	0.904±0.027	0.319±0.024
F4	0.33±0.01	0.329±0.019	0.319±0.014	0.919±0.023

Supplementary Table 3. Description of four signals in Fig. 6C.

	c-DNA-1&2	c-DNA-1&3	c-DNA-1&4	c-DNA-2&3	c-DNA-2&4	c-DNA-3&4
F1	1.015±0.014	0.98±0.011	0.978±0.002	0.065±0.009	0.071±0.011	0.071±0.035
F2	0.988±0.031	0.069±0.023	0.074±0.021	1.017±0.017	0.996±0.029	0.078±0.012
F3	0.075±0.005	1.001±0.011	0.075±0.008	0.958±0.012	0.076±0.022	0.983±0.018
F4	0.075±0.016	0.069±0.014	1.005±0.015	0.074±0.01	1.012±0.028	0.998±0.011

Supplementary Table 4. Description of four signals at 120 min in Fig. 6F, S9C, S10C and S11C.

	cc-DNA-1	cc-DNA-2	cc-DNA-3	cc-DNA-4
F1	0.545±0.005	0.521±0.075	0498±0.011	0.502±0.010
F2	0.456±0.012	0.518±0.005	0.474±0.011	0.477±0.004
F3	0.490±0.035	0450±0.031	0.506±0.014	0.463±0.011
F4	0.501±0.068	0.497±0.020	0.464±0.023	0.519±0.009

Supplementary Table 5. Description of F1 and F2 signals in Fig. 7C, S15B and S16B.

	poly(A)	poly (T)	poly (C)	poly (G)	abasic sites	PT modification
F1	1.236±0.041	0.159±0.028	1.592±0.044	0.88±0.082	0.68±0.021	1.28±0.045
F2	1.233±0.023	0.095±0.02	1.569±0.181	1.031±0.196	0.72±0.038	1.43±0.212

Supplementary Table 6. Description of F3 and F4 signals in Fig. 7E, G, and I.

	cheating dice (A)	cheating dice (T)	cheating dice (C)	cheating dice (G)	cheating dice (empty)	cheating dice (*)
F3	1.025±0.017	0.828±0.022	1.114±0.025	1.005±0.019	0.933±0.018	1.083±0.035
F4	1.005±0.032	0.879±0.008	1.109±0.054	0.975±0.055	0.923±0.023	1.066±0.039
